# Trends and Clinical Outcomes of Anemia in Women of Reproductive Age: A Retrospective Study Across India

**DOI:** 10.7759/cureus.95555

**Published:** 2025-10-28

**Authors:** Sujata Dalvi, Alka Pandey, Rahul Pathak, Shruti Pal

**Affiliations:** 1 Obstetrics and Gynaecology, Nowrosjee Wadia Maternity Hospital, Mumbai, IND; 2 Obstetrics and Gynecology, Lord Buddha Koshi Medical College and Hospital, Saharsa, IND; 3 Medical Affairs, Indchemie Health Specialities Pvt Ltd., Mumbai, IND

**Keywords:** anemia, hemoglobin, ida, iron deficiency, iron deficiency anemia, iron salts, iron supplements, non-heme irons, reproductive age, women

## Abstract

Background

Iron deficiency anemia (IDA) is highly prevalent among women of reproductive age. Despite the wide availability of oral iron formulations, real-world evidence on prescribing practices, treatment outcomes, and patient experiences remains limited. This retrospective, questionnaire-based study evaluated clinical management strategies for anemia in women, focusing on the comparative effectiveness and tolerability of commonly used oral iron salts.

Methods

This retrospective, multicentric, observational study analyzed anonymized electronic medical records (2018-2024) of anemic women aged 15-49 years across diverse Indian regions. A structured digital questionnaire captured patient-reported symptoms, treatment preferences, and management strategies. The impact of oral iron supplements on hemoglobin, hematocrit, ferritin levels, quality of life, and their associated safety profiles was evaluated. Newer-generation iron formulations were excluded to maintain data uniformity.

Results

Participants (ages 15-49 years, mean 32.79) were drawn from all major regions of India, providing a geographically diverse representation. Hemoglobin was the main diagnostic criterion (91.01%). Anemia severity was moderate in 46.62%, mild in 44.01%, and severe in 9.37%, with iron deficiency as the leading cause in 95.80% cases. All iron supplements showed significant improvement (all p < 0.001) in hemoglobin, hematocrit, and ferritin. Ferrous fumarate, the most frequently prescribed iron salt, likely due to its high elemental iron content and convenient dosing regimen, demonstrated significant improvement in hemoglobin levels, increasing from 8.67 ± 1.12 to 11.66 ± 1.18 g/dL. The greatest hemoglobin rise was seen with ferrous fumarate (2.99 ± 1.45 g/dL), followed by ferrous sulfate (2.45 ± 1.29), ferrous* *ascorbate (2.31 ± 1.27), and ferrous* *gluconate (1.98 ± 1.38). Quality of life improved most with ferrous fumarate (90.10% reporting better overall health). Adverse events were reported in 29% of participants, most commonly constipation (39.06%).

Conclusion

This large-scale, real-world analysis reinforces the central role of oral iron salts in managing IDA among women of reproductive age in India. Ferrous fumarate emerged as the most effective formulation, delivering superior hematologic improvements and quality of life gains with an acceptable safety profile. Notably, its efficacy may be further enhanced when combined with absorption-promoting agents such as protein hydrolysates, which improve non-heme iron solubility and bioavailability. These findings underscore the importance of evidence-based, patient-tailored supplementation strategies and highlight the potential of optimizing existing formulations with synergistic enhancers to strengthen national anemia control efforts.

## Introduction

Anemia is characterized by a reduction in the proportion of circulating red blood cells and is not a primary diagnosis but rather a manifestation of underlying pathological conditions. Its onset and severity depend on the cause, rate of progression, and comorbidities, particularly cardiovascular disease [1]. Anemia remains a significant global health concern among women of reproductive age (WRA) (15-49 years), with prevalence nearly four times higher in developing nations compared to developed countries [2].

According to the World Health Organization (WHO), anemia is defined as hemoglobin (Hb) <120 g/L in non-pregnant women and <110 g/L in pregnant women, with severity classified as mild (11.0-11.9 g/dL), moderate (8.0-10.9 g/dL), and severe (<8.0 g/dL) [3]. Globally, anemia affects 30.7% of WRA [4] and approximately 37% of pregnant women [5], impacting nearly 27% of the world’s population overall, with iron deficiency as the leading cause [6-8]. Iron deficiency anemia (IDA), the most common form worldwide and in India, is multifactorial in origin [5-7]. Contributing factors include nutritional deficiencies (iron, folate, vitamin B12, and vitamin A), dietary practices such as high tea/coffee intake, genetic disorders (sickle cell disease, thalassemia, and G6PD deficiency), chronic blood loss, parasitic infestations, and infections such as malaria, HIV, and tuberculosis [8-11].

In India, the National Family Health Survey (NFHS-5) reported that 57% of women aged 15-49 years are anemic, a slight increase from NFHS-4, with severe anemia affecting fewer than 3% [12,13]. IDA is further exacerbated by socioeconomic challenges, low dietary intake of iron-rich foods, poor bioavailability of dietary iron, frequent pregnancies, menorrhagia, repeated lactation cycles, chronic gastrointestinal bleeding, malabsorption syndromes, poor sanitation, and medication use (e.g., non-steroidal anti-inflammatory drugs (NSAIDs), proton pump inhibitors (PPIs)) [14,15].

To address this burden, government programs such as the National Iron Plus Initiative (NIPI, 2013) introduced iron-folic acid supplementation across age groups. However, the persistently high prevalence highlights that supplementation alone is insufficient [16]. In women of reproductive age, anemia is also driven by reproductive and obstetric factors, including heavy menstrual bleeding, expansion of maternal blood volume during pregnancy, and peripartum blood loss, particularly postpartum hemorrhage [5]. Thus, multifaceted strategies including iron supplementation, deworming, management of menstrual disorders, dietary improvement, and enhanced healthcare access are essential [17].

Anemia significantly impacts women’s health, limiting physical capacity, cognitive function, and quality of life, while also reducing economic and social participation [17,18]. During pregnancy, it increases the risk of low birth weight, preterm birth, and maternal mortality [18].

This retrospective, questionnaire-based study explores the prevalence, causes, and management of anemia among WRA across diverse Indian regions. By assessing current strategies such as iron supplementation, medications, and dietary interventions, it aims to identify gaps and provide insights for more effective public health approaches to improve women’s health outcomes.

## Materials and methods

Study design

This was a retrospective, multicentric, observational study conducted at 5778 healthcare centres located in major cities and rural villages across India, including Patna, Kolkata, Bhubneswar, Guwahati, and Gaya in the East, Mumbai, Pune, Nagpur, and Surat in the West, Delhi, Lucknow, and Ghaziabad in the North, and Hyderabad, Bangalore, Chennai, Vijaywada in the South. The study was designed to assess real-world clinical management practices in anemia among WRA.

The study was conducted in compliance with the Declaration of Helsinki and the Indian Council of Medical Research (ICMR) National Ethical Guidelines for Biomedical and Health Research Involving Human Participants (2017). The study protocol and associated documents were reviewed and approved by an independent Institutional Ethics Committee at Lifepoint Multispeciality Hospital, Pune (protocol no: IND/ANM/01; approval date: April 25, 2024). As the study involved retrospective analysis of anonymized patient data, a waiver of informed consent was granted. The anonymized and aggregated data from electronic medical records (EMRs) of Indian patients were collected between May 2024 and March 2025, based on clinical records from January 2018 to December 2024. Patient confidentiality was maintained using anonymized and de-identified data at the source level, with no identifiable information being accessed or recorded.

Study population and eligibility criteria

The study population included women aged 15-49 years with a documented diagnosis of anemia, defined by Hb levels below 12 g/dL, in accordance with WHO guidelines. Participants were stratified into mild, moderate, or severe anemia groups based on initial Hb values. Participants were stratified into severity categories based on Hb levels for non-pregnant women, with mild anemia defined as Hb of 11.0-11.9 g/dL, moderate as Hb of 8.0-10.9 g/dL, and severe as Hb below 8.0 g/dL, consistent with WHO and Indian public health guidelines. Exclusion criteria included pregnant women, WRA with comorbidities that could confound anemia assessment (such as chronic kidney disease, malignancy, or hematologic disorders), and incomplete or missing clinical records. Patients who had received parenteral iron formulations or newer-generation oral iron therapies (e.g., ferric carboxymaltose, iron polymaltose, or sucrosomial iron) were excluded to ensure consistency in treatment exposure and comparability. The sample size was determined based on the availability of eligible patient records that met the inclusion criteria during the study period.

Data collection and variables

Data were captured using a structured digital case report form (CRF) (see Appendices) by physicians across specialties, including gynecology, obstetrics, general medicine, and internal medicine. Variables collected included demographic details (age, clinical setting), menstrual and gynecological history (age at menarche, menstrual patterns), and diagnostic data such as Hb, hematocrit, and serum ferritin levels. Treatment details were recorded, including the type of oral iron salt prescribed (ferrous fumarate, ferrous sulfate, ferrous gluconate, or ferrous ascorbate), dosage, duration of therapy, and adherence status. Prescriptions involving newer-generation iron formulations such as ferric carboxymaltose, iron polymaltose, and sucrosomial iron were excluded to maintain uniformity in the data set. Follow-up hematological parameters and physician-reported treatment response were also collected.

Treatment response was assessed by comparing pre- and post-treatment laboratory values across different iron salt groups. The primary outcome was the change in Hb, hematocrit, and serum ferritin levels. Secondary outcomes included changes in quality of life (QoL) measures and the incidence of adverse events.

Clinical efficacy and QoL assessment

The primary efficacy outcomes included mean changes in Hb, hematocrit, and serum ferritin levels from baseline to post-treatment follow-up. Treatment response was assessed across the different oral iron formulations and stratified by initial anemia severity. Secondary outcomes included patient-reported changes in QoL indicators, which were captured through structured physician-patient feedback recorded in the CRF. QoL parameters, including fatigue, physical activity, emotional well-being, and overall health perception, were assessed.

Safety evaluation

Adverse events (AEs) associated with iron supplementation were systematically extracted from EMRs. Particular attention was paid to gastrointestinal side effects such as nausea, constipation, abdominal discomfort, and metallic taste, which are commonly associated with oral iron salts. The frequency and nature of AEs were analyzed across different iron formulations. Data on treatment discontinuation due to AEs were also recorded to assess the relative tolerability of each formulation.

Statistical analysis

Descriptive statistics were employed to summarize demographics and baseline characteristics. Continuous variables were expressed as means ± standard deviation (SD), and categorical variables as percentages. Paired t-tests were used to assess the significance of changes in hematological parameters from baseline to post-treatment across all groups. Statistical analyses were conducted using XLSTAT v2021.3.1 (Lumivero, LLC, Denver, Colorado, United States) and R v4.0.5 (R Foundation for Statistical Computing, Vienna, Austria, https://www.R-project.org/), with a p-value <0.05 considered statistically significant.

## Results

Patient demographics and baseline characteristics

**Figure 1:**
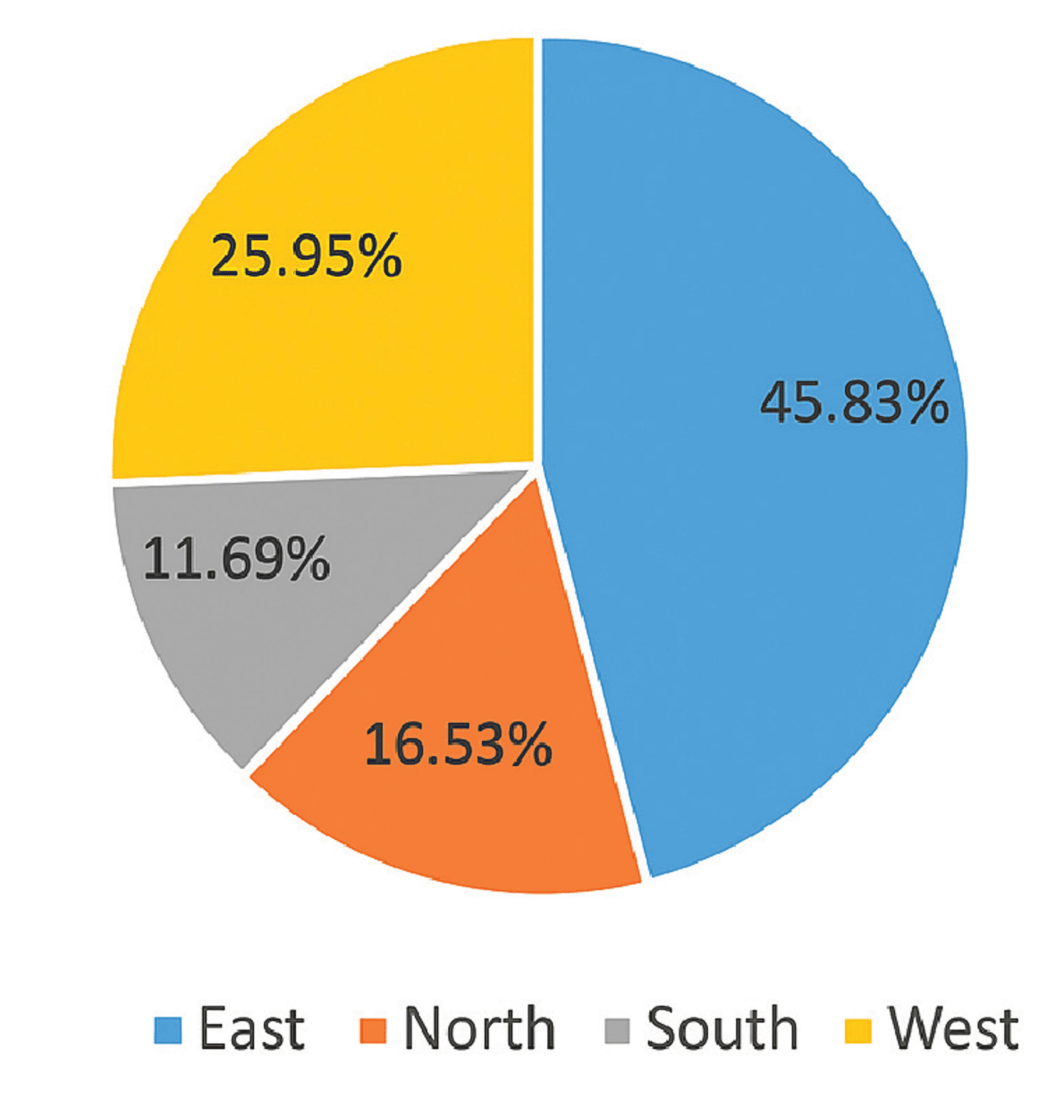
Geographic distribution of women with anemia across Indian regions

A total of 9,283 female patients with anemia were enrolled, with a mean age of 32.79 years (SD ±6.96). Most participants were from eastern India (n=4,256; 45.83%), followed by the west (n=2,409; 25.95%), north (n=1,535; 16.53%), and south (n=1,083; 11.69%)(Figure 1).

Occupation-wise, 4,427 (47.69%) were professionals and 4,356 (46.93%) homemakers. A smaller subset of 366 (3.94%) had a high school education, and 134 (1.44%) were currently in school. At the time of menarche, 5,352 participants (57.65%) were aged ≤14 years, while 3,931 (42.35%) were aged >14 years (Table 1). Among enrolled participants, 3,151 (33.94%) reported irregular menstrual cycles, while 6,132 (66.06%) had regular cycles. Excessive menstrual bleeding was reported by 4,011 (43.21%), of whom 174 (4.35%) experienced it for over a year, 1,392 (34.70%) for 6-12 months, and 2,445 (60.95%) for less than six months (Table 1). Of the 9,283 participants, 8,448 (91.01%) were diagnosed using Hb levels as the primary criterion. Fewer were diagnosed by mean corpuscular volume (n=245; 2.64%), serum ferritin (n=459; 4.94%), or red cell distribution width (n=131; 1.41%) (Table 1).

**Table 1:** Demographic, gynecological, and baseline clinical characteristics of women with anemia (N=9283) Data are expressed as mean ± standard deviation (SD) or n (%)

Parameters	Categories	Values
Age (years)	Mean (±SD)	32.79 (±6.96)
Geographic location	East	4,256 (45.83%)
	West	2,409 (25.95%)
	North	1,535 (16.53%)
	South	1,083 (11.69%)
Occupation	College-going	366 (3.94%)
	Homemaker	4,356 (46.93%)
	Professional	4,427 (47.69%)
	School-going	134 (1.44%)
Age at the time of menarche	≤ 14 years	5,352 (57.65%)
	> 14 years	3,931 (42.35%)
Menstrual cycle	Irregular	3,151 (33.94%)
	Regular	6,132 (66.06%)
Excessive menstrual bleeding	Yes	4,011 (43.21%)
Duration	< 6 months	2,445 (60.95%)
	6 months-1 year	1,392 (34.70%)
	>1 year	174 (4.35%)
Diagnostic criteria used for Anemia	Hemoglobin	8,448 (91.01%)
	MCV	245 (2.64%)
	Serum Ferritin	459 (4.94%)
	RDW	131 (1.41%)

Among the 9,283 patients, 4,328 (46.62%) had moderate anemia, 4,085 (44.01%) had mild, and 870 (9.37%) had severe anemia. Iron deficiency was the leading cause, affecting 8,892 (95.80%) participants. Anemia due to vitamin B12 deficiency was seen in 309 (3.32%), folate deficiency in 40 (0.43%), and hemolytic or sickle cell anemia in 42 (0.45%) (Table 2).

**Table 2:** Prevalence, severity, and etiological distribution of anemia (N=9283)

Parametera	Categories	Frequency (Percentage)
Severity of Anemia	Mild	4,085 (44.01%)
	Moderate	4,328 (46.62%)
	Severe	870 (9.37%)
Type of Anemia	Iron Deficiency Anemia	8,892 (95.80%)
	Vitamin B12 deficiency	309 (3.32%)
	Folate deficiency	40 (0.43%)
	Hemolytic & sickle cell	42 (0.45%)

Management strategies for anemia

The majority of enrolled patients (n =7,435; 80.09%) were treated with iron supplementation. Among these, 433 (5.82%) received ferrous sulfate, 368 (4.95%) received ferrous ascorbate, 80 (1.08%) were treated with ferrous gluconate, and the majority (n=6,554; 88.15%) were prescribed ferrous fumarate, likely due to its high elemental iron content and simple dosing regimen. Dietary modifications were implemented for 833 participants (8.97%), while 557 (6.00%) received other medications. Blood transfusions were given to 311 participants (3.35%), and vitamin B12 supplements were provided to 125 individuals (1.35%) (Table 3). The elemental iron content and dosing frequency vary across different oral iron formulations commonly used in clinical practice, and a summary is provided in Table 4. 

**Table 3:** Clinical management strategies for anemia among study participants (N=7435)

Management strategies	Frequency (Percentage)
Dietary changes	833 (8.97%)
Iron supplements	7,435 (80.09%)
Ferrous fumarate	6,554 (88.15%)
Ferrous sulfate	433 (5.82%)
Ferrous ascorbate	368 (4.95%)
Ferrous gluconate	80 (1.08%)
Vitamin B12 supplements	125 (1.35%)
Folate supplements	22 (0.24%)
Blood transfusion	311 (3.35%)
Other Medications	557 (6.00%)
Herbal/alternative therapies	0%

**Table 4:** Commonly used oral iron formulations: tablet strength, elemental iron content, and recommended adult dosing

Ferrous Salt Form	Formulation (tablet)	Elemental iron	Recommended Adult Dosage
Ferrous fumarate	300 mg	99 mg (33%)	1 tablet, once per day or once every other day
Ferrous sulfate	325 mg	65 mg (20%)	1-2 tablets, once per day or once every other day
Ferrous gluconate	325 mg	39 mg (12%)	1-3 tablets, once per day or once every other day
Ferrous ascorbate	-	100 mg (15%)	1 tablet, once per day or once every other day

Following treatment, a significant improvement was observed in hematological parameters. The mean Hb level increased from 8.65 ± 1.12 g/dL at baseline to 11.58 ± 1.22 g/dL post-treatment. Similarly, the mean hematocrit levels rose from 26.02 ± 4.30% to 32.98 ± 3.21%, and ferritin levels increased from 16.00 ± 6.48 ng/mL to 34.36 ± 4.82 ng/mL. The differences in all three parameters before and after treatment were statistically significant (p < 0.001) (Table 5).

**Table 5:** Hematological parameters (hemoglobin, hematocrit, ferritin) before and after iron therapy Pre- and post-treatment comparisons performed using paired Student’s t-test; significance set at p<0.05.

Parameter	Baseline, mean±SD	Post Treatment, mean±SD	p-value	Change, mean±SD
Hemoglobin (g/dL)	8.65 ± 1.12	11.58 ± 1.22	<0.0001	2.91 ± 1.45
Hematocrit (%)	26.02 ± 4.30	32.98 ± 3.21	<0.0001	6.98 ± 4.46
Ferritin (ng/mL)	16.00 ± 6.48	34.36 ± 4.82	<0.0001	18.58 ± 7.15

Out of the total study population (N = 9,283), 7,435 participants received iron supplementation. The remaining participants either received alternative management (dietary advice, vitamin B12 or folate supplements, or transfusion) or had incomplete post-treatment data. Overall, iron therapy was moderately effective for most participants, 69.04% (5,133 out of 7,435), while only 1.65% (123 participants) found it completely ineffective (Table 6). 

**Table 6:** Overall efficacy of iron therapy and post-treatment quality of life outcomes (N=7435) NOTE: Data were from only participants who received oral iron supplementation (n=7435) and had complete follow-up data. The parameters were derived from two data sources within the same medical record framework. Efficacy data (treatment response and change in hemoglobin) were extracted directly from medical records, based on physician-entered laboratory and follow-up records. Quality-of-life (QoL) data were also captured through the same case report form (CRF). These were not taken through separate surveys but recorded contemporaneously during follow-up visits in the remark section.

Parameter	Categories	Frequency (Percentage)
Efficacy of iron therapy	Not effective	123 (1.65%)
	Somewhat effective	920 (12.37%)
	Moderately effective	5133 (69.04%)
	Very effective	762 (10.25%)
	Extremely effective	497 (6.69%)
Quality of life (post-treatment improvement)	Improved overall health	4478 (60.23%)
	Increased physical activity	1,148 (15.44%)
	Improved emotional health	727 (9.78%)
	Reduced fatigue	1,082 (14.55%)

Comparative efficacy of four oral iron supplements

Hb Restoration

**Figure 2:**
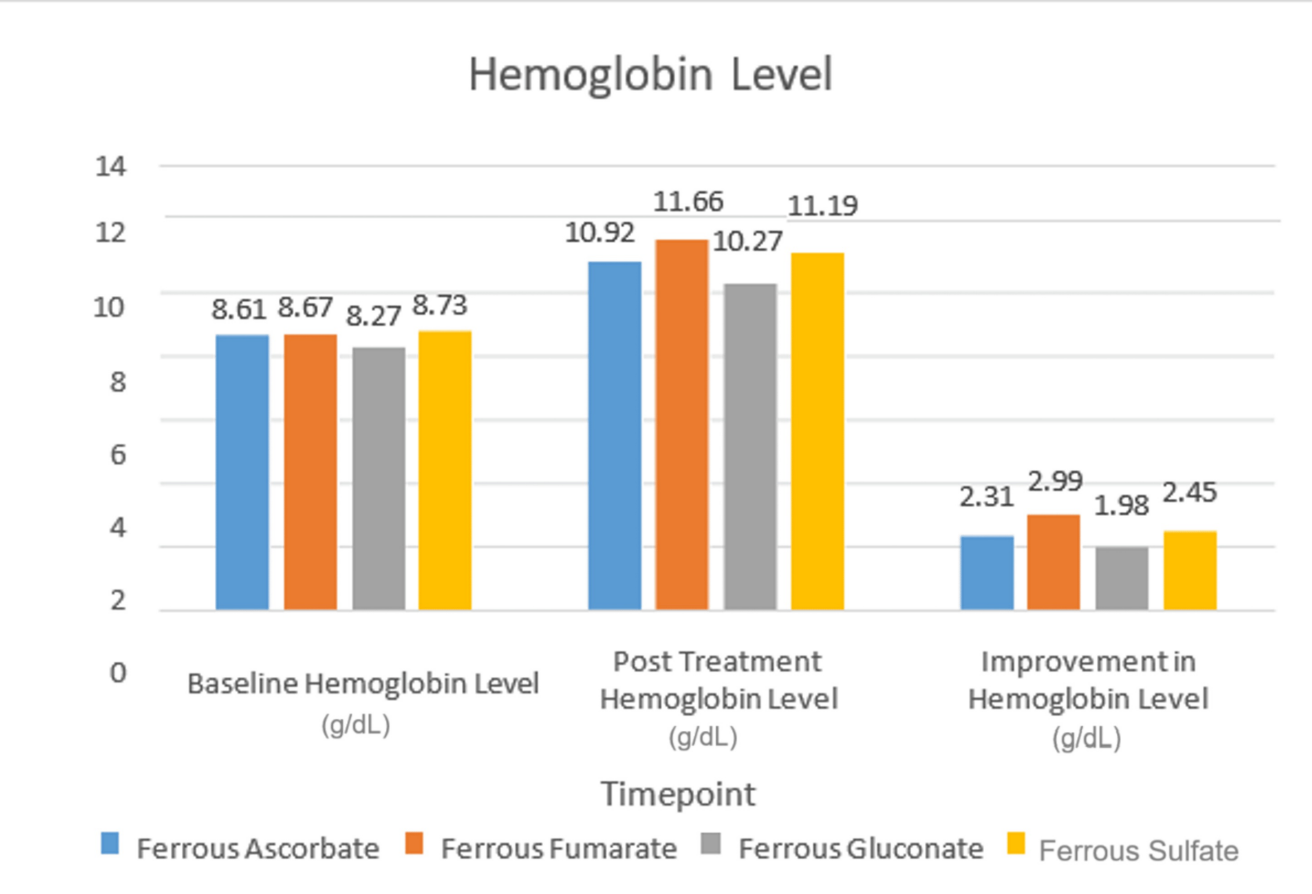
Comparative hemoglobin response following treatment with four oral iron formulations (N=7435) NOTE: This was generated from data of participants who received oral iron therapy and had both baseline and post-treatment hemoglobin records available — a total of 7435 participants.

Hb improvement was assessed across four oral iron supplement groups. In the ferrous ascorbate group, Hb increased from 8.61 ± 1.20 g/dL to 10.92 ± 1.38 g/dL, while in the ferrous fumarate group, it rose from 8.67 ± 1.12 g/dL to 11.66 ± 1.18 g/dL. For ferrous gluconate, Hb levels went from 8.27 ± 1.40 g/dL to 10.27 ± 1.39 g/dL, and in the ferrous sulfate group, the levels increased from 8.73 ± 1.00 g/dL to 11.19 ± 1.21 g/dL. The greatest Hb improvement was seen in the ferrous fumarate group (2.99 ± 1.45 g/dL), followed by ferrous sulfate (2.45 ± 1.29 g/dL), ferrous ascorbate (2.31 ± 1.27 g/dL), and ferrous gluconate (1.98 ± 1.38 g/dL). All groups showed statistically significant increases in Hb (p < 0.001) (Figure 2, Table 7). 

**Table 7:** Comparative hemoglobin improvement across four oral iron formulations SD: Standard deviation

Parameter	Ferrous ascorbate, mean±SD	Ferrous fumarate, mean±SD	Ferrous gluconate, mean±SD	Ferrous sulfate, mean±SD
Baseline hemoglobin (g/dL)	8.61 ± 1.20	8.67 ± 1.12	8.27 ± 1.40	8.73 ± 1.00
Post-treatment hemoglobin (g/dL)	10.92 ± 1.38	11.66 ± 1.18	10.27 ± 1.39	11.19 ± 1.21
Hemoglobin improvement (g/dL)	2.31 ± 1.27	2.99 ± 1.45	1.98 ± 1.38	2.45 ± 1.29

Improvement in QoL

QoL outcomes were assessed across all four oral iron supplement groups. Among patients receiving ferrous fumarate (n=6,554), 90.10% (n=5,905) reported overall health improvement, 84.35% (n=5,528) noted increased physical activity, 89.26% (n=5,850) experienced reduced fatigue, and 90.40% (n=5,925) reported improved emotional well-being. In the ferrous ascorbate group (n=368), 4.46% (n=16) reported improved overall health, 8.51% (n=31) noted increased physical activity, 6.46% (n=24) experienced reduced fatigue, and 4.00% (n=15) reported better emotional well-being. Among those receiving ferrous sulfate (n=433), 4.76% (n=21) reported improved overall health, 5.73% (n=25) noted increased physical activity, 2.92% (n=13) experienced reduced fatigue, and 3.84% (n=17) reported better emotional well-being. For patients treated with ferrous gluconate (n=80), 1.25% (n=1) reported improvement in overall health, 1.25% (n=1) noted increased physical activity, 1.25% (n=1) experienced reduced fatigue, and 1.25% (n=1) reported improved emotional well-being.

Safety Outcome of Oral Iron Supplements

A total of 2,683 AEs were reported, accounting for 29% of the study population. Constipation was the most commonly reported AE, occurring in 1,048 patients (39.06%), followed by black stools in 621 patients (23.15%) and nausea in 410 patients (15.28%). Additional side effects included abdominal discomfort or pain in 258 patients (9.62%), diarrhea in 151 patients (5.63%), and heartburn in 128 patients (4.77%) (Table 8).

**Table 8:** Distribution of adverse effects of iron therapy (N=2683)

Categories	Frequency (Percentage)
Constipation	1048 (39.06%)
Diarrhea	151 (5.63%)
Nausea	410 (15.28%)
Abdominal discomfort/pain	258 (9.62%)
Heartburn	128 (4.77%)
Metallic Taste	57 (2.12%)
Black stools	621 (23.15%)
Hypersensitivity reactions	0 (0.00%)
Hypotension	0 (0.00%)
Others	10 (0.37%)

## Discussion

Anemia is one of the world’s leading causes of health burden, making it one of the most urgent global public health issues [19]. In the current retrospective study, we collected data from medical records and information from participants regarding their experiences with anemia and its management via digital questionnaires. Hb was used as a marker in most participants to detect anemic conditions. Other markers, such as MCV, serum ferritin, and RDW, were used in very few patients. The age range of the patients included in the study was 15-49 years. The maximum number of anemic WRA (45.81%) included in the study were from eastern India, followed by the western region (26.00%). Northern and southern India contributed less compared to the other two. Overall, the WRA were suffering from either mild or moderate anemia. Very few (9.37%) were suffering from severe anemia. A greater number of WRA reported menarche before the age of 14, which may partly explain the high prevalence of anemia observed. Early menarche increases the duration of menstrual blood loss during adolescence, thereby elevating iron requirements and susceptibility to anemia. This is consistent with findings from a rural Indian study that reported a higher prevalence of anemia among girls with earlier menarche [20]. Additionally, a Chilean cohort found that lower dietary iron intake was associated with earlier onset of menarche, suggesting a bidirectional relationship between nutrition and menarche timing [21].

Although 66.06% of WRA in the study had regular menstrual cycles, a significant proportion experienced excessive menstrual bleeding, primarily for less than six months, followed by six months to one year. This aligns with Jha and Rao (2019), who found that even short-term menorrhagia can lead to significant iron loss and increase the risk of anemia [22]. Munoz et al. (2011) emphasized that prolonged bleeding can contribute to chronic IDA, highlighting the need for early management of menstrual irregularities [23].

In the current study, only 1.44% WRA were school-going, and 3.94% were high school-going. The remaining were all professionals or homemakers. The majority of them were suffering from anemia due to iron deficiency. The three markers used for anemia showed low Hb, hematocrit, and ferritin levels at baseline. Studies have shown that women with little to no education, as well as those with only secondary-level education, are more likely to experience anemia compared to women with higher levels of education. Education is strongly correlated with income and wealth and plays a crucial role in shaping health behaviors. Educated individuals tend to adopt healthier lifestyles, including better nutrition, improved health decision-making, and enhanced hygienic practices, all of which contribute to better health outcomes [2,24].

IDA is the most prevalent form of anemia, a finding that was also observed in our study. This aligns with previous research, which highlights iron deficiency as the leading cause of anemia in various populations [25]. Oral iron replacement therapy is a simple and effective approach for treating iron deficiency, except in cases of non-adherence, intolerance, unresponsiveness, or severe anemia [26]. In one retrospective study, the significant increase in Hb and iron parameters across all treatment regimens after one month of therapy also corroborates findings from existing studies [27].

Ferrous salts are traditionally recommended, and several bivalent iron salts have been used for supplementation. In our study, we compared the efficacy of four iron salts, ferrous ascorbate, fumarate, gluconate, and sulphate, in terms of change in Hb levels and QoL pre- and post-treatment. Traditionally, the recommended daily dose is 100-200 mg iron per day, given as three or four divided doses of ferrous salts [28]. While the 100 mg guideline is standard, individualized dosing strategies based on patient response are recommended. In our study, participants received a daily dose of elemental iron between 100 mg and 130 mg for an average of 2.86 months (±2.42) based on the severity of anemia. The ferrous fumarate group showed significantly higher Hb levels, indicating that higher doses, combined with an appropriate iron salt, may enhance treatment outcomes.

The majority of the WRA were on ferrous fumarate, and the Hb levels in the ferrous fumarate group were significantly higher compared to the other three groups. However, the levels post treatment were statistically significant compared to the baseline levels. All aspects of QoL were improved in a significant number of WRA on ferrous fumarate. Our study results are supported by a few other studies. Mehta, in a 2003 report, described a cohort of 27 patients with iron deficiency anaemia who exhibited no hematologic response following treatment with iron polymaltose complex (IPC) administered over 4-52 weeks [29]. Notably, all patients demonstrated a favourable response upon subsequent administration of ferrous fumarate for durations ranging from 4 to 13 weeks. Consistent findings were reported by Ruiz-Argüelles et al., who documented that 75 out of 240 patients (31%) diagnosed with iron deficiency anaemia and treated with oral IPC at their institution failed to achieve an adequate response [30]. At the time of study enrollment, these non-responders had a median Hb concentration of 10.3 g/dL. Following treatment with oral ferrous fumarate for periods spanning 1-14 months, the median Hb levels significantly increased to 12.5 g/dL (P < 0.01), underscoring the superior efficacy of ferrous fumarate in this patient subset. Overall, in the current study, iron therapy was found to be moderately effective in the majority of the patients.

Our study evaluated four non-heme iron salts, ferrous ascorbate, fumarate, gluconate, and sulfate, whose absorption is influenced by dietary factors. Non-heme iron has lower and more variable bioavailability than heme iron and is affected by inhibitors (e.g., phytates, calcium) and enhancers like ascorbic acid [31]. Despite this, ferrous fumarate showed greater efficacy in improving Hb levels. This suggests that factors such as higher elemental iron content, individual response, or better gastrointestinal tolerance may also contribute to treatment outcomes [32,33], highlighting the complex interplay between iron formulation and absorption.

Protein hydrolysates, particularly those derived from milk casein and soy, have emerged as effective promoters of iron absorption due to their ability to chelate minerals and enhance their bioavailability. These hydrolysates contain peptides that not only keep iron soluble and reduce ferric iron to the more absorbable ferrous form but also facilitate transport across intestinal membranes. Structural properties such as peptide size, amino acid composition, and the presence of specific residues influence their capacity to bind iron and enhance its uptake in the gut. Calcium-binding phosphopeptides (CPP), derived from casein, have shown significant iron-binding capabilities and play a key role in improving iron absorption both in vitro and in vivo, as well as in animal models [34,35].

Combining ferrous fumarate with casein protein hydrolysate offers synergistic benefits, such as improved iron absorption and increased Hb synthesis, potentially enhancing muscle function, energy levels, and immune response. Studies have shown that bioactive peptides derived from casein and soy protein hydrolysates can significantly improve non-heme iron bioavailability, making them suitable co-supplements with ferrous fumarate [34,36]. Furthermore, the use of multi-amino acid iron chelates (IMAACs) has been demonstrated to enhance Hb levels, supporting the potential of protein hydrolysates as iron absorption enhancers [35]. These findings suggest that the addition of protein hydrolysates to iron supplements represents a promising strategy to combat iron deficiency and optimize iron status. Based on the evidence presented, the improved outcomes observed in this study with ferrous fumarate supplementation can be attributed to the synergistic effect of protein hydrolysates, which enhance iron solubility, stability, and intestinal absorption. In addition, the inclusion of multiple micronutrients such as folic acid, vitamin B12, vitamin B6, calcium, copper, and zinc alongside iron supplementation may marginally enhance Hb synthesis and improve response compared with iron supplementation alone. These findings suggest that the superior performance of ferrous fumarate over other iron salts may not solely be due to the salt itself, but rather to its enhanced bioavailability when combined with protein hydrolysates and complementary micronutrients, making this combination a promising strategy for optimizing iron supplementation [37].

Oral iron supplements are known to cause more gastrointestinal side effects than intravenous iron or placebo [37]. In our study, 2,683 adverse events were reported, with 29% of participants experiencing at least one. The most common adverse effects were constipation, black stools, and nausea; importantly, these were generally mild and self-limiting.

Given its superior efficacy in our study, ferrous fumarate, with its unique formulation, plays a significant role in managing iron deficiency anemia. The formulation’s high elemental iron content (~33%) allows for greater iron delivery per dose, making it an effective option for improving Hb levels [32]. Unlike other iron salts, ferrous fumarate’s stability and bioavailability are enhanced, providing a more efficient means of replenishing iron stores. This, along with its good tolerability, positions ferrous fumarate as a promising treatment, particularly in patients requiring higher doses to address iron deficiency [33]. Its formulation combines both efficacy and patient adherence, making it a preferred option in clinical practice.

This study has some limitations. The retrospective design and absence of randomization may introduce bias, limiting causal conclusions. The short follow-up period (2.4 months) restricts the assessment of long-term outcomes. Importantly, there was an unequal distribution of participants across treatment groups, with ferrous fumarate being disproportionately represented. This imbalance limits the strength of direct comparisons between salts and may introduce bias. Excluding pregnant women helped reduce confounding but limits generalizability to that group. Newer iron formulations, though known for better gastrointestinal tolerability and absorption, were not included in this study due to their limited usage in the retrospective dataset. As a result, the findings may not be generalizable to all currently available iron therapies. Despite these limitations, the study’s strengths include its large, diverse sample and comparison of commonly used anemia treatments in real-world settings. These findings offer practical insights into clinical practice.

## Conclusions

This large, multicenter retrospective study reaffirms the central role of oral iron supplementation in improving hematologic outcomes and quality of life among anemic WRA in India. Among the various iron formulations evaluated, ferrous fumarate emerged as the most effective, demonstrating the highest increase in Hb levels and a superior impact on overall well-being, physical activity, and fatigue reduction. Its favorable safety profile, along with its high elemental iron content and good gastrointestinal tolerability, makes it an especially valuable option in routine clinical practice. Notably, ferrous fumarate’s efficacy may be further enhanced when administered alongside absorption promoters such as protein hydrolysates, which are known to improve the solubility and bioavailability of non-heme iron. Incorporating such synergistic compounds into iron supplementation regimens holds promise for overcoming limitations in iron absorption, especially in populations consuming predominantly plant-based diets with high levels of inhibitors like phytates.

These findings support a shift toward evidence-based, tailored supplementation strategies that prioritize both efficacy and absorption efficiency. As India continues to grapple with a high burden of IDA, particularly among women, standardizing the use of clinically validated formulations like ferrous fumarate, potentially in combination with iron absorption enhancers, may significantly improve treatment outcomes. Digital tools and community health programs can further aid in delivering personalized care, ensuring adherence, and minimizing anemia’s long-term health and socioeconomic consequences.

## Figures and Tables

**Figure 3:**
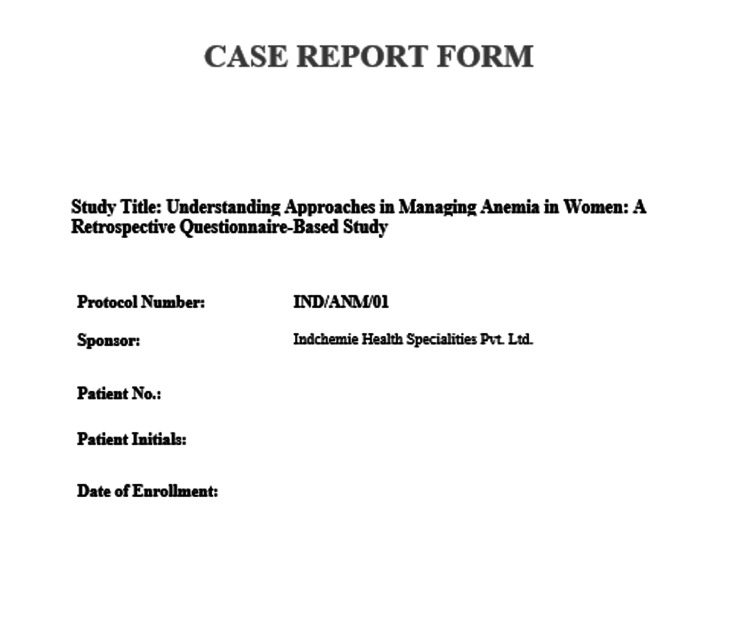
Page 1 of the case report form

**Figure 4:**
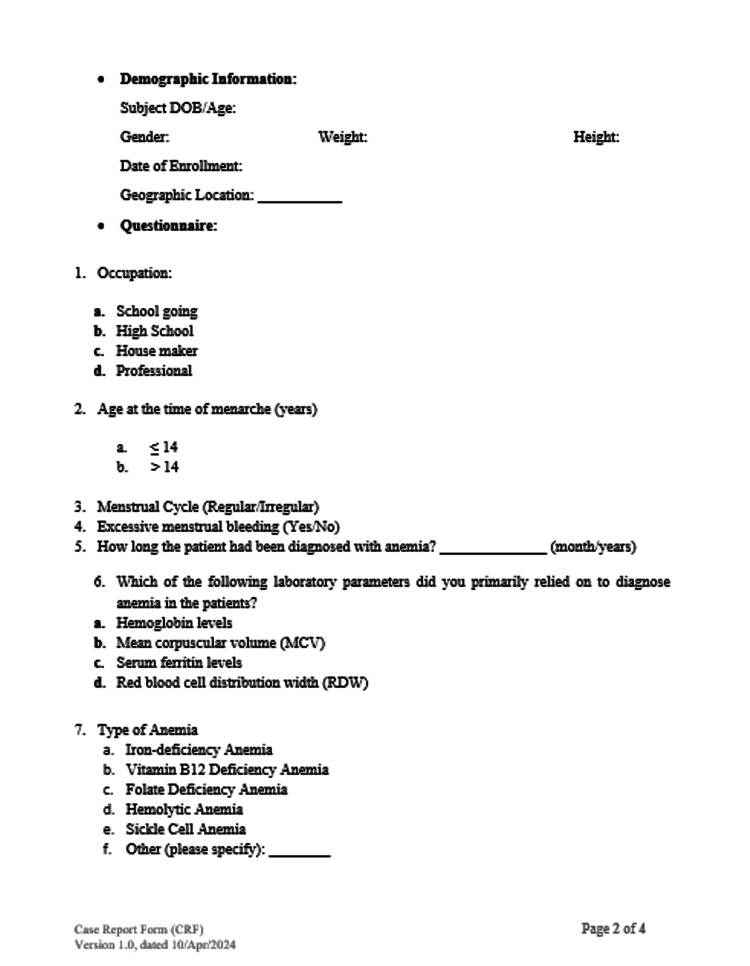
Page 2 of the case report form

**Figure 5:**
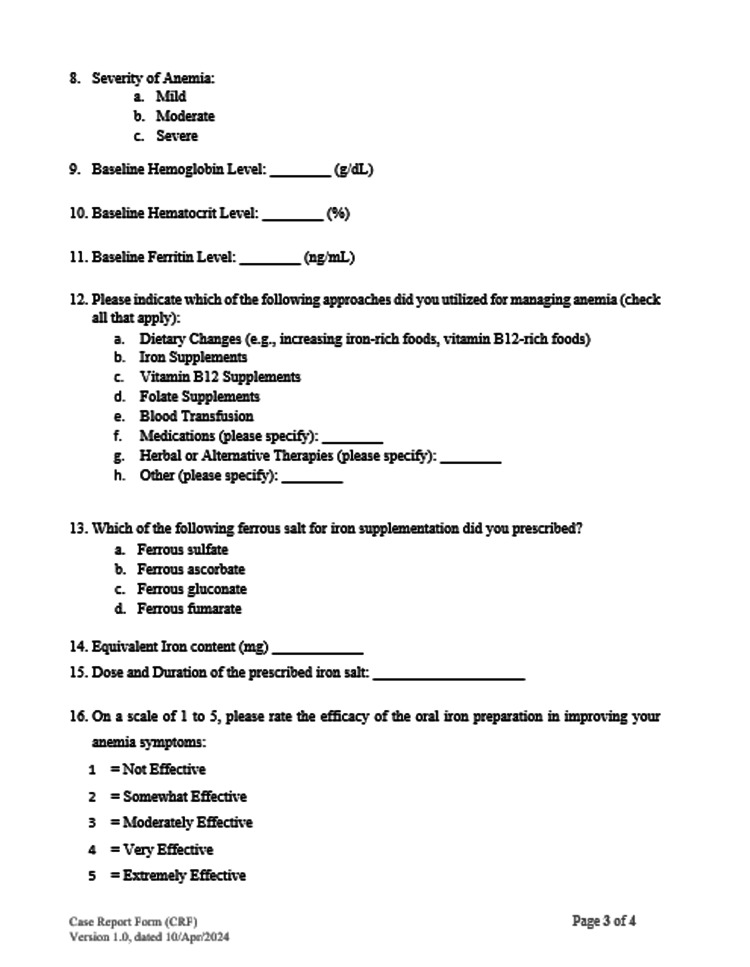
Page 3 of the case report form

**Figure 6:**
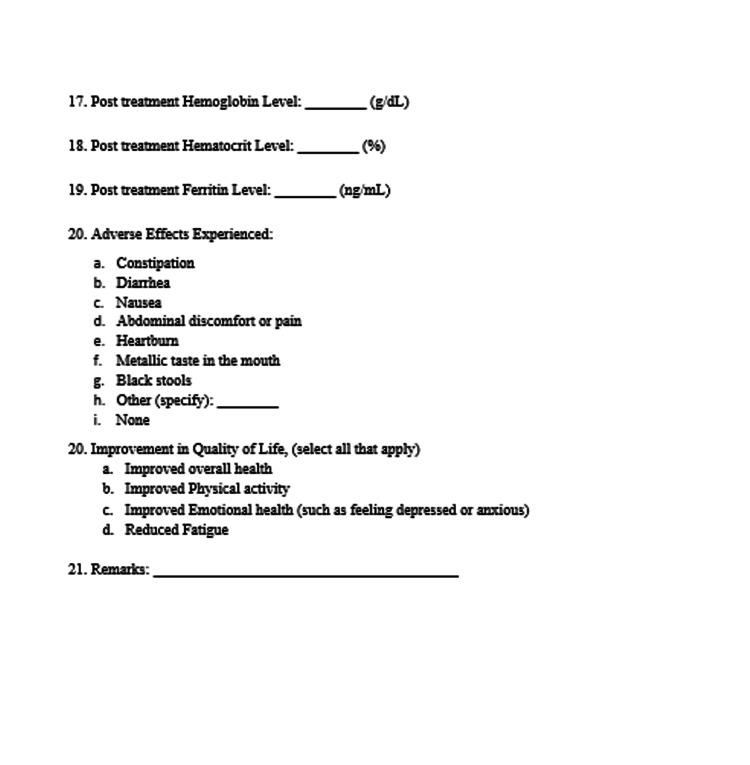
Page 4 of the case report form
